# Emergence of Epidemic *Neisseria meningitidis* Serogroup X Meningitis in Togo and Burkina Faso

**DOI:** 10.1371/journal.pone.0019513

**Published:** 2011-05-20

**Authors:** Isabelle Delrieu, Seydou Yaro, Tsidi A. S. Tamekloé, Berthe-Marie Njanpop-Lafourcade, Haoua Tall, Philippe Jaillard, Macaire S. Ouedraogo, Kossi Badziklou, Oumarou Sanou, Aly Drabo, Bradford D. Gessner, Jean L. Kambou, Judith E. Mueller

**Affiliations:** 1 Agence de Médecine Préventive, Paris, France; 2 Centre Muraz, Bobo-Dioulasso, Burkina Faso; 3 Ministry of Health, Lomé, Togo; 4 Centre Hospitalier Universitaire Souro Sanou, Bobo-Dioulasso, Burkina Faso; 5 Institut National de l'Hygiène, Lomé, Togo; 6 Ministry of Health, Ouagadougou, Burkina Faso; Health Protection Agency, United kingdom

## Abstract

Serogroup X meningococci (*Nm*X) historically have caused sporadic and clustered meningitis cases in sub-Saharan Africa. To study recent *Nm*X epidemiology, we analyzed data from population-based, sentinel and passive surveillance, and outbreak investigations of bacterial meningitis in Togo and Burkina Faso during 2006–2010. Cerebrospinal fluid specimens were analyzed by PCR. In Togo during 2006–2009, *Nm*X accounted for 16% of the 702 confirmed bacterial meningitis cases. Kozah district experienced an *Nm*X outbreak in March 2007 with an *Nm*X seasonal cumulative incidence of 33/100,000. In Burkina Faso during 2007–2010, *Nm*X accounted for 7% of the 778 confirmed bacterial meningitis cases, with an increase from 2009 to 2010 (4% to 35% of all confirmed cases, respectively). In 2010, *Nm*X epidemics occurred in northern and central regions of Burkina Faso; the highest district cumulative incidence of *Nm*X was estimated as 130/100,000 during March–April. Although limited to a few districts, we have documented *Nm*X meningitis epidemics occurring with a seasonal incidence previously only reported in the meningitis belt for *Nm*W135 and *Nm*A, which argues for development of an *Nm*X vaccine.

## Introduction

In the African meningitis belt, *Neisseria meningitidis* serogroup A (*Nm*A) is associated with seasonal hyperendemic and epidemic meningitis, but other meningococcal serogroups, pneumococci, and *Haemophilus influenzae* type b contribute to the meningitis burden. In 2002 for example, *Nm*W135 was responsible for an epidemic wave in Burkina Faso with about 13,000 cases [1], and the same sequence type ST-11 was found as the main agent during an outbreak in southern Niger during 2009 [2].

During the last two decades, sporadic cases of serogroup X (*Nm*X) meningitis have occurred throughout the meningitis belt. Niger reported cases during 1990 [3] and 1997, with the detection of 83 confirmed cases in Niamey [4]. In northern Ghana during 2000, 7 *Nm*X cases and a concomitant increase of *Nm*X carriage were reported [5], [6]. In 2006, from January to June, an outbreak with over 550 *Nm*X cases occurred in the south-western region of Niger including Niamey [7]. During the same year, 11 *Nm*X were reported in north-eastern Uganda [8] and 5 in the bordering districts of Kenya [9]. Nevertheless, *Nm*X has not been documented to cause outbreaks of the magnitude previously associated with *Nm*A and *Nm*W135.

While some meningococcal polysaccharide or conjugate vaccines now include serogroup W135, no vaccine currently exists against serogroup X. Some studies to define natural immunity to *Nm*X [Amadou et al., 17^th^ International Pathogenic *Neisseria* Conference; Banff, Canada; 2010 Sep 11–16; Abstract P108] and to identify correlates of protection and purify the polysaccharide (G. Norheim, personal communication) are ongoing, but no larger development project for an affordable vaccine exists. Lack of an *Nm*X vaccine would be less relevant if hyperendemic cases and smaller outbreaks occurred only sporadically. However, a vaccine for epidemic response or prevention would be needed if *Nm*X caused epidemics similar to *Nm*A or *Nm*W135. To address this issue, we analyzed incidence, microbiological and clinical data to determine *Nm*X burden and epidemiological patterns in Burkina Faso and Togo during 2006–2010.

## Methods

### Ethics statement

The decision to perform lumbar punctures was made by the treating health care providers. The national Burkina Faso and Centre Muraz ethics committees approved the Bobo-Dioulasso surveillance study protocol, for which informed written consent was obtained from participants, as we collected individual data beyond that obtained for routine national surveillance. All other surveillance activities were part of the national surveillance systems and were conducted with authorization from the Togo and Burkina Faso Ministries of Health; as these systems were part of routine public health surveillance, ethical committee approval and informed consent were not sought.

### Surveillance activities

**Figure 1:**
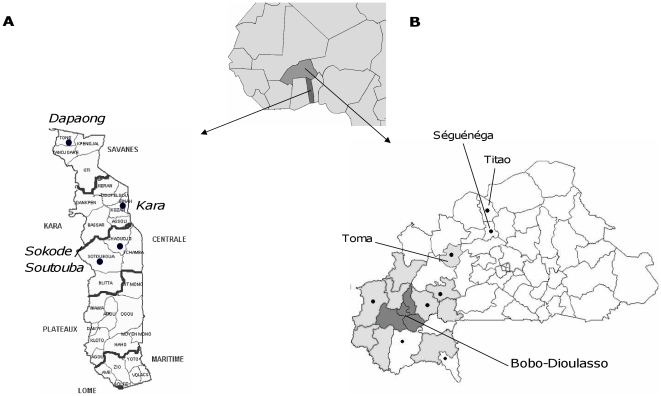
Surveillance systems in Togo and Burkina Faso. A. Togo: sentinel surveillance sites in Dapaong, Kara, Sokodé and Soutouba during 2006–2009. B. Burkina Faso: 2009–2010 passive surveillance in the western country (light grey); 2007–2010 surveillance study (dark grey); 2007–2010 mobile laboratory outbreak investigations (dark dots).

We used data from surveillance activities in Togo and Burkina Faso (**Figure 1**), based on polymerase chain reaction (PCR) as the principal diagnostic technique. Activities were implemented in collaboration with local Ministries of Health.

In Togo, January 2006–September 2009, we conducted sentinel surveillance in major referral hospitals situated in Tône district (regional hospital of Savanes region), Kozah district (regional hospital of Kara region), Tchaoudjo district (regional hospital of Central region) and Soutouba district (district hospital) [10] (**Figure 1A**). Each site had a theoretically covered population of 150,000 to 300,000 (in total about 15% of the Togolese population). Togo is situated at the southern border of the meningitis belt and, while the Savanes region in the north has a long dry season from November to May with hyperendemic meningitis incidence, Kara and Centrale region have a less pronounced dry season depending on inter-annual variations of the inter-tropical convergence zone [11]. All persons presenting with suspected acute bacterial meningitis at one of the sentinel sites received a lumbar puncture. The number of cases included during the sentinel surveillance was about 50% higher than the number of cases routinely reported. Cerebrospinal fluid (CSF) was evaluated by culture and by PCR.

In Burkina Faso, we conducted a surveillance study in 4 sanitary districts (865,000 inhabitants, about 6% of the Burkinabè population), around Bobo-Dioulasso March 2007–December 2009. As previously published [12], all suspected meningitis patients presenting at any health center or hospital received a lumbar puncture and the corresponding CSF was analyzed by culture, latex agglutination and PCR. The number of cases included during the surveillance study was nearly 20% higher than the number of cases routinely reported in these districts.

In western Burkina Faso, passive surveillance has been conducted since February 2009 in 10 districts (**Figure 1B**). We provided assistance so that all health centers of the participating districts could send CSF samples from all suspected meningitis cases for PCR analysis. The theoretically covered population was 2,397,200 (15.5% of the total Burkinabè population). We evaluated 35% of routinely reported cases in these districts.

Lastly, outbreak investigations to define meningitis etiology were conducted throughout Burkina Faso using a mobile laboratory equipped with bacteriology facilities for immediate analysis [13]. CSF samples were also analysed by PCR. Twenty-two outbreak investigations were thus conducted among 78 epidemic district declarations during 2007–2010.

For all surveillance activities, we used the WHO case definition for suspected bacterial meningitis (sudden onset of fever >38.5°C rectal and meningeal signs such as neck stiffness, bulging fontanel, convulsion, vomiting or other) [14]. Lumbar puncture was performed by the treating nurse or physician in the health center or hospital. Patient age and CSF appearance were available from all surveillance systems. Information on clinical outcome (death, severe complications during hospitalization such as shock or coma, and persistent motor-sensorial deficit at hospital discharge) was available from Togo and the surveillance study around Bobo-Dioulasso.

Small targeted preventative vaccination campaigns with meningococcal serogroup A/C polysaccharide vaccine were performed in Savanes and Kara regions (Togo) during 2006–2010, and reactive mass campaigns using the same vaccine in Burkina Faso during 2006–2008 to cover all districts of the country.

### Laboratory analysis

All bacteriological analyses were performed by standard methods [14]. A multiplex technique was used for PCR analyses at Centre Muraz in Bobo-Dioulasso [12], which involved gene amplification of *crg*A for *Nm*, *bexA* for *Haemophilus influenzae* (*Hi*), *lytA* for *Streptococcus pneumoniae* (*Sp*), *siaD* for *Nm* genogroups B, C, Y, X, and W135, and *mynB* for *Nm*A. The WHO Collaborating Center at the Institut de Médecine Tropicale du Service de Santé des Armées (IMTSSA, France) performed phenotyping and multi-locus sequence typing as previously described [15] on a selection of 28 *Nm* isolates and on CSF from 10 persons with *Nm*X meningitis, as no *Nm*X isolates had been stored.

### Data analysis

All statistical analyses were performed using STATA 10.0 (StataCorp, College Station, TX, USA). We calculated cumulative weekly, monthly and seasonal incidences as number of reported or confirmed cases for specific weeks, months or the total meningitis season (January through May), divided by the population estimated provided by the sanitary authorities for the surveyed population. For Séguénéga district, we calculated serogroup-specific district incidence from the incidence of suspected cases reported by Ministries of Health multiplied by the percentage of *Nm*X among all suspected cases identified by laboratory analyses in the sentinel sites. 95%-confidence intervals (95%-CI) to these estimates were calculated using the formula Var(X,Y)  =  Var(X) Var(Y) + Var(X) E[Y]^2^ + E[X]^2^ Var(Y) for the multiplication of variances. To compare the association of outcomes with epidemiological characteristics of cases, we defined a variable encompassing five categories based on the serogroup and epidemiological context (e.g., “epidemic *Nm*A”, “non epidemic *Nm*A”, “*Nm*X Centrale” for cases from outside the meningitis belt).The epidemic context in the districts was defined based upon national reporting and the WHO thresholds for epidemics [14]. We tested for differences in case characteristics between serogroups X and A using the Chi-square test and an alpha error of 5%. In addition, we performed multivariate logistic regression analyses to estimate the association of serogroup X or A meningitis with (i) age group, controlling simultaneously for sex, epidemic situation and country; and (ii) death or sequelae, controlling simultaneously for age group, sex, epidemic situation, and CSF aspect (Togo only). Multivariate logistic regression analyses included suspected cases identified in sentinel surveillance in Togo, the Bobo-Dioulasso surveillance study and passive surveillance in western Burkina Faso. Results are reported as odds ratios (OR) with 95% confidence interval (95%-CI).

## Results

### Togo 2006–2009

**Figure 2:**
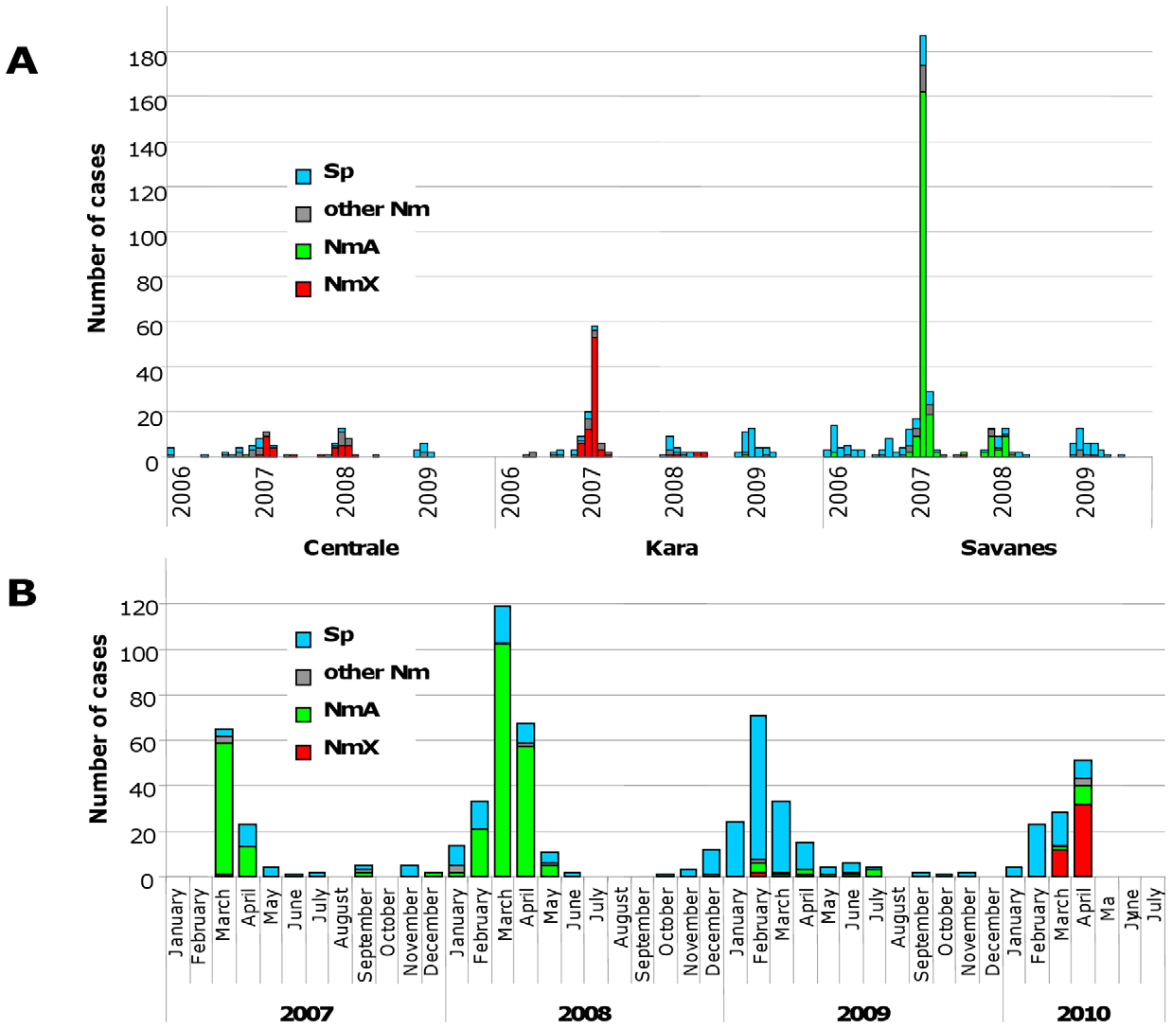
Number of confirmed bacterial meningitis cases, by etiology. A. Togo: sentinel hospital-based surveillance in three sanitary regions 2006–2009. B. Western Burkina Faso: exhaustive and passive surveillance and outbreak investigations, 2007–2010. *Sp: Streptococcus pneumoniae*, *Nm: Neisseria meningitides*.

During the surveillance period, 1,376 suspected cases were identified, of which 1,044 (76%) were tested by PCR. A bacterial etiology was confirmed for 702 (51%), of which 428 (61%) were *Nm*. Overall, *Nm*A and *Nm*X accounted for 31% (N = 219) and 16% (N = 114) of confirmed cases, respectively, and this figure varied by region and year (**Figure 2A**). Within sites and years, *Nm*A and *Nm*X did not occur simultaneously. Four percent of *Nm*X and 18% of *Nm*A cases occurred in a non-epidemic context.

During the 2007 meningitis season, Tône and Kozah districts declared epidemics. In Tône district (281,303 inhabitants), among 316 suspected and 258 confirmed bacterial cases, 196 (76%) were identified as *Nm*A, 32 (12%) *Sp*, 12 *Nm*W135, 9 non-groupable *Nm*, 8 *Hi* and 1 *Nm*X (**Figure 2A**). The peak monthly *Nm*A cumulative incidence per 100,000 was 49 (95%-CI, 41–58) during March and the seasonal incidence 70 (95%-CI, 60–80). In Kozah district, (224,398 inhabitants), among 172 suspected and 97 confirmed bacterial cases, 75 were *Nm*X (77%), 8 *Nm*W135 (8%), 7 *Sp* (7%), 5 non-groupable *Nm* (5%), 2 *Hi* (2%) and none *Nm*A (**Figure 2A**). The *Nm*X peak monthly incidence per 100,000 was 24 (95%-CI, 18–31) during March and the *Nm*X seasonal incidence 33 (95%-CI, 26–42).

**Figure 3:**
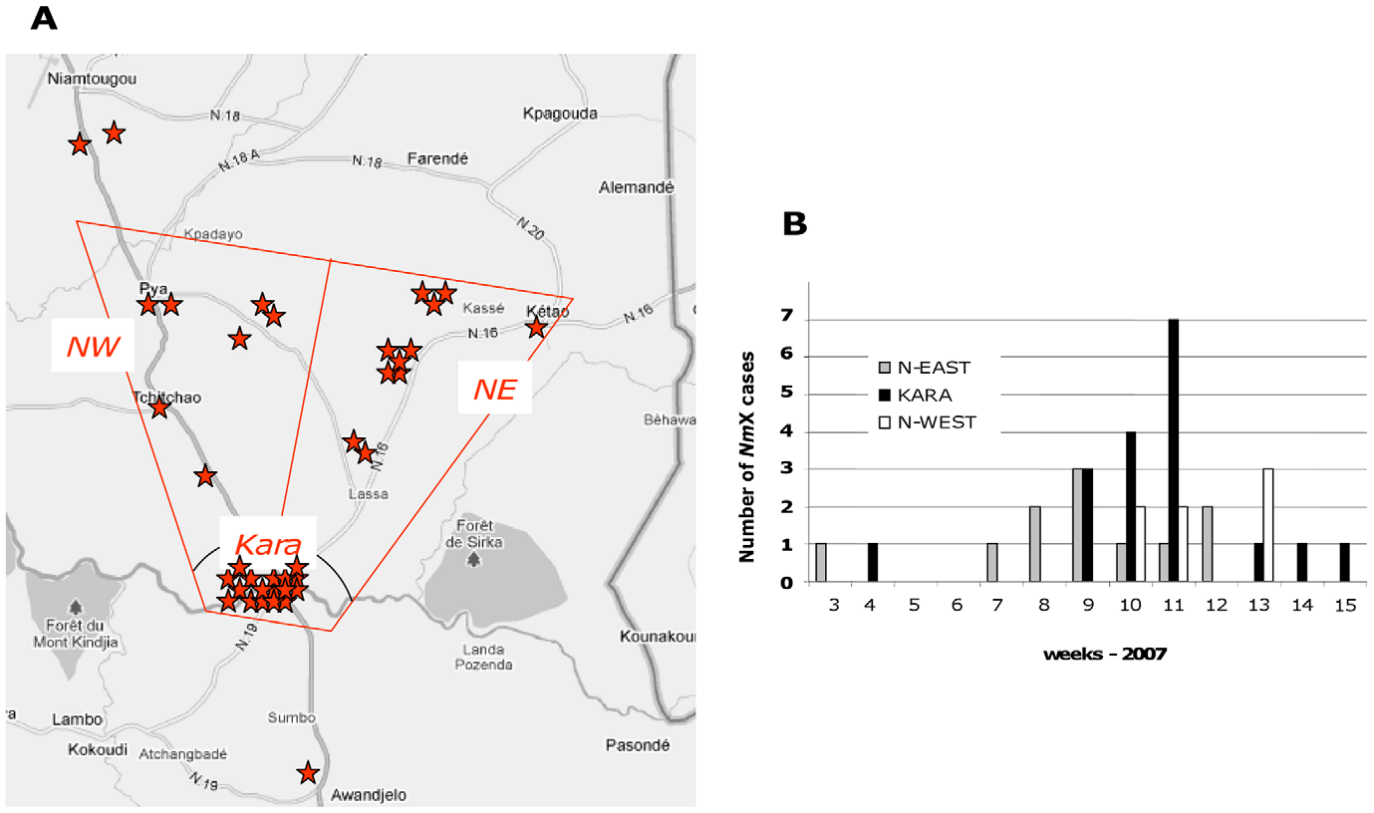
Spatio-temporal epidemiology of the NmX outbreak in the Kozah district, Togo, 2007. A. Geographical distribution of 39 *Nm*X cases with information on patient residence. Three zones were determined: Kara City, North West of Kara City (NW) and North East of Kara City (NE). Each red star represents one confirmed *Nm*X case. B. Weekly identification of *Nm*X cases, from January to June 2007 (36 cases with information available on both patient residency and month of CSF collection). *Nm: Neisseria meningitides*.

In the Kozah *Nm*X outbreak, based on 46 persons with *Nm*X meningitis and accurate information on residence, 78% of cases (N = 36) were localized within an approximately 60 km^2^ triangle, bounded by Kara City and the 2 main roads going north (**Figure 3**). Three overlapping waves of *Nm*X occurrence could be described: first in north-eastern Kozah district from week 3 to 12 (N = 11), then in Kara City which reported half of the total cases mostly during week 9 to 11 (N = 18), and lastly in north-western Kozah district from week 10 to 13 (N = 7).

During the following 2 years, no meningitis epidemic was reported in Savanes or Kara regions. During this period, in the Savanes region, *Nm*A accounted for 22 cases (69% of all *Nm* detected cases), whereas 9 (28%) were *Nm*W135 and only one *Nm*X case was observed. In the Kara region, six of the 15 *Nm* cases were *Nm*X, 3 were *Nm*W135 and 1 *Nm*A.

In the Centrale region, which is situated outside the meningitis belt, 24 and 25 *Nm* cases were seen during the 2007 and 2008 seasons, respectively, with 15 *Nm*X cases each year. No *NmA* case and only rare sporadic *Nm*W135 cases were detected throughout the entire surveillance period (**Figure 2A**).

### Burkina Faso 2007–2010

#### Population-based surveillance

In the Bobo-Dioulasso surveillance study, a total of 1,008 suspected cases were included during March 2007–December 2009, with 976 CSF analyzed. Among 455 cases with confirmed bacterial etiology (47% of all suspected cases) (**Figure 2B**), 286 (63%) were due to *Nm*, 159 (35%) to *Sp* and 9 (2%) to *Hi*. Of the 286 *Nm* cases, 269 (94%) were *Nm*A, 9 (3%) non-groupable *Nm*, 5 (2%) *Nm*W135, 2 (1%) *Nm*X and 1 *Nm*C (<0.5%).

#### Passive surveillance

During 2009–2010, 633 CSF were collected and 203 (32%) confirmed for a bacterial etiology. During 2009, 80 (87%) of 92 confirmed cases were *Sp*, 6 (7%) *Nm*A, 4 (4%) *Hi*, one (1%) non-groupable *Nm* and one (1%) *Nm*X, while in 2010, of 111 confirmed cases, *Nm*X accounted for 44 (40%), *Sp* for 52 (47%), *Nm*A for 9 (8%), *Nm*W135 for 4 (4%) and *Hi* for 2 cases (2%).

#### Outbreak investigations

For 49 (16%) of the 301 collected CSF, an etiology was confirmed by PCR. During 2007 and 2008, among the 83 confirmed cases, 79 (95%) were *Nm*A and 4 (5%) *Sp*. During 2009, among 9 confirmed cases with etiologic confirmation, 6 were *Nm*X, 2 *Sp* and 1 *Nm*A. During the 2010, 59 CSF were collected in western and northern regions; of 28 confirmed cases, 5 (18%) were *Nm*X, 21 (75%) *Nm*A, 2 (7%) non-groupable *Nm* and none *Sp*.

**Figure 4:**
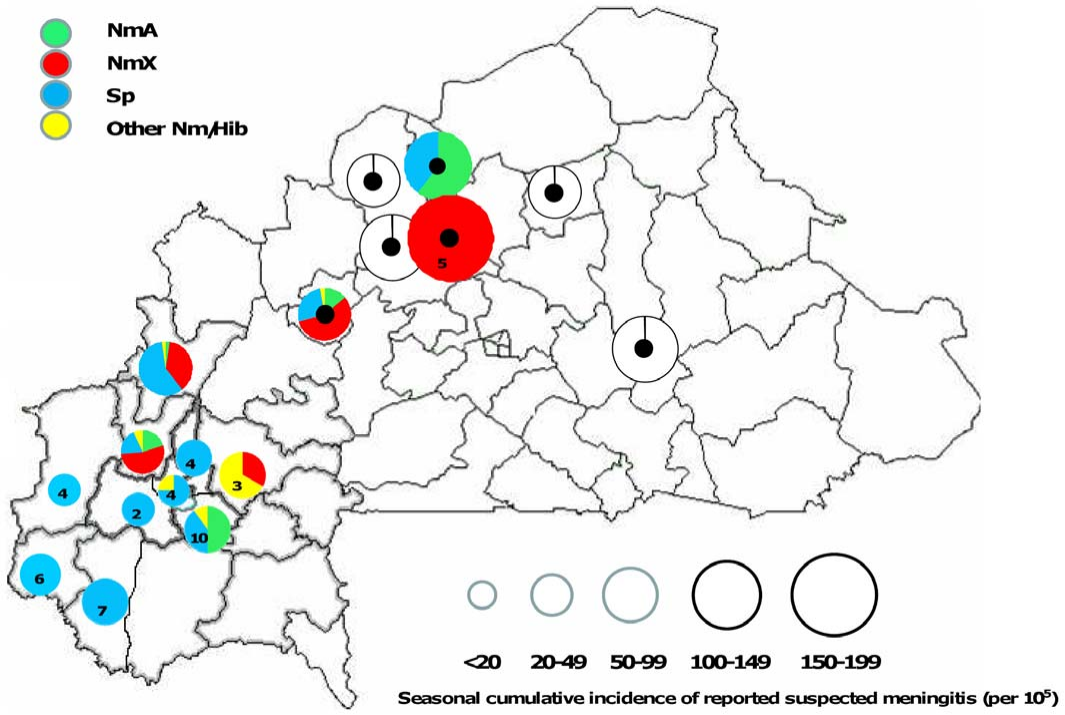
District level etiology distribution among PCR-confirmed bacterial meningitis cases from surveillance in Burkina Faso, 2010. Circle size is proportional to the seasonal cumulative incidence in the districts (January–June). Black spots indicate districts with reported epidemics during 2010. The white colored circles are epidemic districts for which the etiology distribution is not available for this report. Numbers in circles represent the total number of confirmed cases in epidemic districts if the total number was less than 15.

In Séguénéga district in northern Burkina Faso, April 2009, CSF was collected from 12 suspected cases, of which 5 were *Nm*X and 1 *Sp*. During March-April 2010, this district reported 259 suspected cases for 179,200 inhabitants, corresponding to a cumulative incidence of 145/100,000. Of six suspected cases evaluated during week 11 (34 cases reported by the national surveillance), five were *Nm*X and one had no etiology confirmed. Based on these data, we estimated a cumulative *Nm*X incidence of 120 (95%-CI, 75–166) per 100,000 during March–April and a weekly *Nm*X incidence of 16 (95%-CI, 8–24) per 100,000 during week 11. In addition to Séguénéga, seven other Burkina Faso districts declared an epidemic during 2010, in two of which our surveillance activities allowed to identify the causative agent: in Toma district we detected 21 *Nm*X, 5 *Nm*A and 10 *Sp* among 74 suspected cases, while in Titao (district, neighboring Séguénéga) we detected 21 *Nm*A and 1 *Sp* among 49 suspected cases **(** **Figure 4**).

Overall, during 2007 to 2010, *Nm*X accounted for 7% (N = 57) of the 778 confirmed cases, with an increase from 2009 (N = 7; 4% of all confirmed cases) to 2010 (N = 49; 35% of all confirmed cases). Twenty eight percent of *Nm*X and 59% of *Nm*A cases occurred in a non-epidemic context.

### Characteristics of NmX cases

Information on age and sex was available for 168 *Nm*X and 530 *Nm*A cases, and on clinical outcomes at hospital discharge for 98 *Nm*X and 426 *Nm*A cases (**Table 1**). Age and sex distribution did not differ significantly between *Nm*X and *Nm*A cases in comparable epidemic contexts. Across serogroups, epidemiological contexts and countries, children between 5 and 14 years of age contributed most to meningococcal disease burden (36%–77% of cases). In Burkina Faso during epidemics, females were under-represented among *Nm*X compared to *Nm*A cases (27% vs. 56%, *P* = 0.019), but this was not observed in Togo. In both settings, *Nm*X cases tended to present less frequently with visibly purulent CSF aspect (*Nm*X vs. *Nm*A, 81.1% vs. 91.6%, *P*<0.000). In multivariate analyses among all suspected cases, compared to 5- to 14-year-old children, diagnosis of *Nm*X was less likely in infants <1 year (OR 0.30, 95%-CI 0.88–1.03) and young adults 15–29 years of age (OR 0.55, 95%-CI 0.31–0.97), while diagnosis of *Nm*A was more likely in these age groups (OR 3.28, 95%-CI 0.96–11.26 for infants and OR 1.83, 95%-CI 1.03–3.27 for young adults).

**Table 1:** Epidemiological characteristics of *Nm*A and *Nm*X confirmed cases of meningitis, by country.

			*Nm*A (all)	*Nm*X (all)	*Nm*A epidemic	*Nm*X epidemic	*Nm*A non epidemic	*Nm*X non epidemic	*Nm*X Centrale
Female: % (N)		BF	39 (222)	46 (114)	38 (181)	49 (75)	41 (41)	50 (8)	39 (31)
		T	41 (309)	28 (57)	56 (39)	27 (26)	39 (270)	29 (31)	n/a
Age group (years): % (N)	<1	BF	9 (221)	2 (114)	8 (180)	1 (75)	12 (41)	12(8)	0 (31)
		T	5 (310)	2 (54)	0 (39)	0 (26)	6 (271)	4 (28)	n/a
	1–4	BF	17 (221)	15 (114)	17 (180)	15 (75)	17 (41)	25 (8)	13 (31)
		T	21 (310)	19 (54)	23 (39)	15 (26)	20 (271)	21 (28)	n/a
	5–14	BF	45 (221)	49 (114)	47 (180)	51 (75)	37 (41)	36 (8)	48 (31)
		T	52 (310)	72 (54)	64 (39)	77 (26)	50 (271)	68 (28)	n/a
	15–29	BF	22 (221)	18 (114)	22 (180)	16 (75)	20 (41)	25 (8)	23 (31)
		T	19 (310)	7 (54)	13 (39)	8 (26)	20 (271)	7 (28)	n/a
	≥30	BF	8 (221)	16 (114)	6 (180)	17 (75)	15 (41)	0 (8)	16 (31)
		T	3 (310)	0 (54)	0 (39)	0 (26)	4 (271)	0 (28)	n/a
Visibly purulent CSF: % (N)		BF	93 (216)	86 (112)	92 (177)	84 (75)	100 (39)	71 (7)	93 (30)
		T	91 (311)	71 (52)	95 (39)	71 (21)	90 (272)	71 (31)	n/a
Severe complication or motor-sensorial deficit *: % (N)		BF	12 (198)	7 (96)	10 (177)	4 (75)	29 (21)	25 (4)	18 (17)
		T	6 (230)	0 (2)	5 (19)	n/a	6 (211)	0 (2)	n/a
Death: % (N)		BF	10 (214)	16 (108)	8 (177)	11 (74)	16 (37)	17 (6)	29 (28)
		T	10 (268)	0 (2)	5 (20)	n/a	10 (248)	0 (2)	n/a

BF is for Burkina Faso data; T is for Togo data. *Nm: Neisseria meningitis.*

*Severe complication: coma or shock during hospitalisation; motor-sensorial deficit as observed at hospital discharge.

Information on clinical outcomes of both *Nm*A and *Nm*X cases was available in substantial number mostly from Togo. No significant difference was seen between *Nm*X and *Nm*A for severe complications or motor-sensorial deficit (7% vs. 12%, P = 0.206), and fatal outcome tended to occur more frequently in *Nm*X than *Nm*A cases (16% vs. 10%, P = 0.120). Compared to *Nm*X cases occurring in an epidemic context from other regions in Togo, *Nm*X cases in the Centrale region were characterized by higher case fatality (28.6% vs. 10.8%, *P* = 0.028) and more frequent severe complications or motor-sensorial deficit (17.7% vs. 4.0%, *P* = 0.040). In general, irrespectively of serogroup or country, cases occurring during epidemics tended to be associated with lower case fatality and less severe outcome than those that occurred in a non-epidemic context. In multivariate analyses including all *Nm*A and *Nm*X cases confirmed in Togo, compared to *Nm*A, serogroup X caused substantial but insignificant negative association with observation of severe complications or motor-sensorial deficit (OR 0.41, 95%-CI 0.13–1.28), and none with mortality (OR 1.01, 95%-CI 0.42–2.42). Among *Nm*X cases, fatal outcome was associated with persons aged 30 years or older (OR 4.89, 95% CI 2.00–11.92 compared to age 5–14 yrs).

### Phenotype and sequence type

Multi-locus sequence typing described ST-181 for all 10 CSF samples tested from the Kozah outbreak. One *Nm*X isolate from Bobo-Dioulasso, 2007, was X:NT: P1.5, ST-181. Among 48 *Nm*A isolates tested (found in Togo and Bobo-Dioulasso, 2007–2008), 45 were A:4:P1.9, ST-2859. The remaining three, all found in Bobo-Dioulasso, 2008, were a new sequence type ST-6968 (single-locus variant of ST-2859). One *Nm*W135 isolate found in Bobo-Dioulasso, 2007–2008, was W135:NT:P1.5,2, ST-2881.

## Discussion

This analysis of surveillance data from Togo and Burkina Faso during 2006–2010 confirms that *Nm*X not only causes small outbreaks and sporadic meningitis cases during seasonal hyper-endemicity, but has epidemic potential. In two districts of Burkina Faso during 2010 and one in Togo 2007, *Nm*X was the predominant etiology, representing 56% of laboratory-confirmed bacterial meningitis cases, while incidences were above the epidemic threshold [14]. In Burkina Faso during 2010, the *Nm*X cumulative incidence during March-April was 120 (95%-CI, 75–166) per 100,000 in the Séguénéga district, and the seasonal cumulative incidence likely was even higher. Previously observed district *Nm*X seasonal cumulative incidences per 100,000 were 28 (95%-CI, 24–31) in Niamey/Niger [7] and 33 (95%-CI, 26–42) in Kozah district/Togo. Consequently, the current report represents the first *Nm*X epidemic with a seasonal incidence comparable to *Nm*A epidemics previously observed in western Burkina Faso [e.g., Secteur 15 district 2006 with 110 (95%-CI, 100–121) per 100,000, AMP/Centre Muraz authors, unpublished data] or Togo [Tône district 2007 with 70 (95%-CI, 60–80) per 100,000], where eventually appropriate reactive vaccine mass campaigns were launched for epidemic control. Although data were not available for this report, *Nm*X was identified as the epidemic agent in three additional districts of Burkina Faso that declared an epidemic in 2010 and *Nm*X cases were found in 15 districts, of which 10 were characterized by complete absence of *Nm*A cases during this season [Delrieu et al., 17^th^ International Pathogenic *Neisseria* Conference; Banff, Canada; 2010 Sep 11–16; abstract OM11]. The 2010 *Nm*X epidemics occurred in a few districts, and not in the form of a geographically expansive epidemic wave spanning several regions or countries. By contrast, *Nm*A has historically caused large epidemics, as did *Nm*W135 during 2002. As with any other serogroups, it is unknown which factors would allow *Nm*X to cause such epidemic waves [16]. If *Nm*X epidemics occurred regularly or covered larger regions, reactive or preventive vaccination could be deemed efficient from the public health perspective. Our analysis did not provide evidence for substantial differences in demographic characteristics or clinical outcomes between *Nm*A and *Nm*X cases, therefore preventive strategies against *Nm*X should target the same groups as against *Nm*A or *Nm*W135.

The *Nm*X sequence type (ST-181) that was responsible for the Kozah outbreak has been identified in the meningitis belt for several decades, first in carriage in Mali in the 1970s [17] and in invasive isolates in Niger, 1997 [4]. The majority of *Nm*X invasive isolates from West Africa since 2000 belonged to this sequence type [17], [18], which more recently was found in carriage in Bobo-Dioulasso in 2008 (X:15:P1.6, ST-198) [Njanpop-Lafourcade et al., 17^th^ International Pathogenic *Neisseria* Conference; Banff, Canada; 2010 Sep 11–16; abstract P051]. ST-751, a genetically related sequence type [5], was found in sporadic cases and carriage in Bobo-Dioulasso during 2003 [15]. However, the longstanding and continuous presence of ST-181 *Nm*X strains in the meningitis belt, especially during recent years, with and without occurrence of epidemics, suggests that factors other than strain virulence are involved in the occurrence of *Nm*X epidemics [19].

The finding that in 2010, Séguénéga district experienced an *Nm*X epidemic while the epidemic in neighboring Titao district was due to *Nm*A, suggests that clonal waves of meningococcal strains, as described in northern Ghana [6], can be specific to relatively small populations. The highly localized dynamic of the Kozah outbreak also lends some support to this hypothesis. Competition between colonizing meningococcal strains may play a role in such variations and may, in addition, define which serogroup will be predominant during an epidemic, if several strains are circulating in the population. This may explain why in all districts during epidemics observed so far (Togo, Burkina Faso, Niger), incidence was high for only a single serogroup at a time. The widespread reactive mass campaigns with serogroup A/C polysaccharide vaccine in Burkina Faso is unlikely to have played a role in the serogroup distribution during epidemics, as polysaccharide vaccines probably lack impact on meningococcal carriage [20]. To better understand the relation between epidemic serogroups, further research should include surveillance at the health center level during epidemics, ideally with frequent multi-locus sequence typing of isolates and carriage studies from a larger area.

To eliminate epidemic *Nm*A meningitis in sub-Saharan Africa, a meningococcal serogroup A conjugate vaccine (MenAfriVac) is being introduced in Burkina Faso, Mali and Niger [21], [22]. Our data have several implications for the impact assessment of this vaccine which currently is designed to use national surveillance data from Burkina Faso. However, the low contribution of *Nm*A to meningococcal disease during recent compared to previous years, occurring already before vaccine introduction, will make it difficult to quantify the impact of group A conjugate vaccine on incidence or case counts, and only data from exhaustive long-term laboratory surveillance comparing the previous to the coming decade may allow an assessment. Our finding of localized *NmX* epidemics underlines the need for widespread, not only sentinel, laboratory surveillance with systematic serogrouping of meningococcal cases, including identification of serogroup X. Continuous high-quality laboratory surveillance over several years also is necessary to survey for serogroup replacement. Since serogroup A conjugate vaccine is expected to impact meningococcal carriage [23], introduction of a group A conjugate vaccine could select for the spread of other *Nm* serogroups, although the currently infrequent colonization with *Nm*A [15] suggests that the risk for replacement is low.

Some conclusions from the presented analysis may be limited by small numbers of cases evaluated during outbreak investigation. Most importantly, the incidence estimation for the Séguénéga epidemic in 2010 is based on laboratory evaluation of six suspected cases. However, additional comparable surveillance data from the Pediatric University Hospital Charles-de-Gaulle, Ouagadougou suggest a similar proportion of suspected cases being *Nm*X (49 *Nm*X among 66 suspected cases, 74%) [Delrieu et al., 17^th^ International Pathogenic *Neisseria* Conference; Banff, Canada; 2010 Sep 11–16; abstract OM11]. Other conclusions may be biased because surveillance activities only included limited time periods and some activities were passive or targeted outbreak evaluations. However, appropriate laboratory methods had been used throughout the reported period and for most activities, including outbreak investigations, data from at least three observation years were available. Also, previous reports from this decade described similarly low incidences of *Nm*X cases [7], [10], [18], [24]. We acknowledge that the interpretation of data from such a variety of surveillance systems is difficult. However, we estimate that these data are the most exhaustive and precise information available from the two countries. The local operational and financial constraints are such that one unified surveillance system with exhaustive laboratory analysis in a wide geographic area has not existed during past years and is challenging to establish.

In summary, our study showed that *Nm*X can cause both hyperendemic and epidemic meningitis in the meningitis belt, while its potential to cause large epidemic waves remains unclear. This underlines that despite ongoing serogroup A conjugate vaccine introduction in some countries, control and prevention of substantial meningitis epidemics due to any serogroup will require ongoing quality surveillance, better understanding of the epidemic meningitis phenomenon, continued vaccine development and support to case management.
